# Predictors of symptom improvement in patients with chronic coronary syndrome after percutaneous coronary intervention

**DOI:** 10.1007/s00392-024-02552-w

**Published:** 2024-10-01

**Authors:** Michael Wester, Franziska Koll, Mark Luedde, Christoph Langer, Markus Resch, Andreas Luchner, Karolina Müller, Florian Zeman, Michael Koller, Lars S. Maier, Samuel Sossalla

**Affiliations:** 1 University Heart Centre Regensburg, Department of Internal Medicine II, University Hospital Regensburg, Franz-Josef-Strauß-Allee 11, 93053 Regensburg, Germany; 2 Cardiologicum Bremerhaven, Bremerhaven, Germany; 3 Kardiologisch-Angiologische Praxis, Heart Centre Bremen, Bremen, Germany; 4 Department of Internal Medicine I, St. Josef Hospital, Regensburg, Germany; 5 Department of Cardiology, Hospital Barmherzige Brüder Regensburg, Regensburg, Germany; 6 Centre for Clinical Studies, University Hospital Regensburg, Regensburg, Germany; 7 Department of Cardiology, University Hospital Giessen and Kerckhoff Heart Centre, Department of Cardiology, Bad Nauheim; Justus-Liebig University of Giessen and German Centre for Cardiovascular Research (DZHK), Partner Site Rhine-Main, Frankfurt Am Main, Germany

**Keywords:** PCI, CCS, Quality of life, Outcome

## Abstract

**Background:**

Decreases in symptom load and improvements in quality of life are important goals in the invasive treatment of symptomatic chronic coronary syndrome (CCS). To date, it is not known which patients profit most from the invasive treatment.

**Methods:**

This sub-analysis of the prospective, multi-centre PLA-pCi-EBO trial includes 145 patients with symptomatic CCS and successful PCI. The prespecified endpoints angina pectoris and quality of life (Seattle Angina Questionnaire–SAQ) were assessed 1 and 6 months after PCI. Predictors of symptom improvement were analyzed by logistic regression analysis.

**Results:**

Quality of life, physical limitation, and angina frequency markedly improved 6 months after PCI. Worse baseline health status (i.e., low SAQ subscales) was the best predictor of highly clinically relevant improvements (≥ 20 points in SAQ subscales) in symptom load and quality of life. Demographic factors (age, sex, body-mass index) and cardiovascular disease severity (number of involved vessels, ejection fraction) did not predict relevant improvements after PCI. The influence of psychologic traits has not previously been assessed. We found that neither optimism nor pessimism had a relevant effect on symptomatic outcome. However, patients who exercised more after PCI had a much larger improvement in quality of life despite no differences in physical limitation or angina frequency.

**Conclusion:**

PCI effectively reduces symptom load and improves quality of life in patients with symptomatic CCS. Reduced baseline health status (symptom load, quality of life) are the only relevant predictors for improvements after PCI. Physical activity after PCI is associated with greater benefits for quality of life.

**Trial registry:**

The German Clinical Trials Register registration number is DRKS0001752.

## Introduction

The high incidence and potentially disabling symptom load of chronic coronary syndromes (CCS) represents a highly relevant health care problem [1]. Pivotal trials in the last 2 decades have challenged the belief in the benefit of percutaneous coronary intervention (PCI) for these patients [2–4]. However, PCI can provide quick and lasting symptom relief in many patients [5]. The optimal patient selection for either medical or invasive treatment remains a matter of debate. As invasive treatment does not provide a clear reduction in mortality over that observed for medical therapy, the effects on symptom load and quality of life become relatively more important [1]. Despite this, most trials focus on treatment effects on “hard endpoints” like death or hospitalization. This poses an important gap in knowledge about a highly relevant disease.

Previous studies that investigated possible predictors for symptomatic outcome included patients with both acute and chronic coronary syndrome which is an inhomogeneous collective as it ranges from stable and asymptomatic patients to patients with acute infarction. Spertus et al. found that in 1518 patients undergoing PCI (patients with acute myocardial infarction were excluded) baseline severity of symptom load was the strongest predictor of improvement in quality of life 1 year after the procedure. Demographics (age, race, sex), clinical characteristics (cardiovascular and non-cardiovascular comorbidities), and disease-severity characteristics (number of diseased vessels, ejection fraction) had negligible effects on changes in quality of life after PCI. The strongest predictor was baseline physical function and angina frequency, meaning that patients with the worst baseline could expect the largest improvements [6]. Arnold et al. investigated 2573 patients undergoing PCI (stable angina pectoris [AP], unstable AP, NSTEMI [non-ST elevation myocardial infarction]; excluding STEMI [ST elevation myocardial infarction]). They found that the majority of patients (76%) were angina free 6 months after PCI [7]. Predictors of persisting angina were lower age, self-reported avoidance of care due to cost, depression, the number of antianginal medications, and extreme pain/discomfort together with lower quality of life at the time of PCI [7]. Zhang et al. looked for predictors of symptom improvement in the COURAGE trial population [8]. Among 1,476 patients with stable AP, they found that the chances of good/excellent physical limitation were increased by higher age, better baseline Seattle Angina Questionnaire (SAQ) physical limitation scores, non-smoking, and lower body-weight [8]. The Chinese PEACE study, which examined 1,611 patients undergoing elective PCI without acute myocardial infarction [9], also found that the strongest predictors of being angina free one year after PCI were better baseline values for angina frequency and quality of life [9]. Collison et al. looked for predictors of post-PCI angina in 230 patients undergoing PCI for chronic or acute but medically stabilized coronary syndromes [10]: predictors of angina three months after PCI were higher body-mass index, current smoker, atrial fibrillation, and previous PCI [10]. In contrast to the patients in those studies, cardiologists are frequently sought out by patients with symptomatic CCS and recommendations for this specific and relevant patient group are not clear. In addition, psychologic influences and patient believes have not been studied at all in these analyses.

The aim of this sub-study of the PLA-pCi-EBO trial was therefore to analyze possible predictors of improvement of symptom load and quality of life in patients with symptomatic CCS after PCI.

## Methods

### Study population

The PLA-pCi-EBO trial is a prospective randomized controlled trial designed to investigate the additional effect of visual demonstration of successful PCI on quality of life and AP in patients with CCS. The study protocol has been published previously [11]. The primary endpoint of this study was the change in quality of life as assessed with the SAQ from baseline to follow-up. Secondary endpoints were changes in the other SAQ-derived scores (physical limitation, angina stability, angina frequency, treatment satisfaction). The results of these primary analyses have been published [12]. As there was no difference in outcomes between randomization arms [12], we examined the whole study population for the current analysis.

Briefly summarized, between April 2019 and September 2020, consecutive symptomatic patients undergoing PCI at five academic centres and large community hospitals in Germany were screened for eligibility. The main inclusion criteria were: age ≥ 18 years, symptomatic coronary artery disease, Canadian Cardiovascular Society angina score ≥ 2, AP frequency ≥ 2/week, and successful implantation of ≥ 1 coronary artery stent, i.e., complete revascularization of the culprit lesion. The main exclusion criteria were: concomitant disease causing dyspnea or chest pain (i.e., left ventricular ejection fraction < 35%; anemia; severe pulmonary disease; severe valvular disease); conditions that prevented sufficient understanding of the visual demonstration and explanation of the angiographic results (language barrier; impaired vision or hearing; dementia).

The study complied with the Declaration of Helsinki. Each study site obtained approval by the local ethics committee (reference number 19-1261-101) and all patients provided written informed consent for participation. The German Clinical Trials Register registration number is DRKS00017524.

### Patient-reported outcome measures

Patient-reported symptom burden was evaluated using the SAQ [13] at the time of hospital admission for PCI and at the follow-up visits 1 and 6 months after the procedure. The SAQ consists of the five subscales “quality of life (disease perception)”, “physical limitation”, “angina frequency”, “angina stability”, and “treatment satisfaction”. The scales range from 0 points (worst symptoms) to 100 points (no symptoms). We concentrated our current analysis on the clinically most relevant subscales “quality of life”, “physical limitation “, and “angina frequency”. Changes in the angina stability subscale are difficult to interpret and to compare between groups, as the subscale itself measures a change over time. The “treatment satisfaction” subscale showed very high initial values and only minimal change over time [12]. This may be attributed to the timing of the questionnaire during the hospitalization for PCI when the patient receives maximum treatment and medical attention, which would boost treatment satisfaction and thus poses a high risk of bias.

To aid in the clinical interpretation of the SAQ scales, they may be categorized into four ranges of scores (quartiles): 0–24 indicates a very poor to poor health status; 25–49 indicates a poor to fair health status; 50–74 indicates a fair to good health status; and 75–100 indicates a good to excellent health status [14].

It can be challenging to unequivocally classify patients’ symptoms. Coronary artery disease can cause typical and atypical chest pain and present as a manifold of symptoms, including but not limited to dyspnea, unusual fatigue, and generalized weakness [1, 15]. In addition, these symptoms are often concomitant to varying degrees. Our study included patients who presented with typical angina (i.e., pain, pressure, or tightness in the chest during physical exercise or emotional stress). However, in some patients this was not their main symptom: in some cases dyspnea or sharp pain was more dominant and was the limiting symptom. These patients were labelled as “atypical angina”.

We used the German version of the revised LOT-R to evaluate optimism and pessimism [16]. The LOT-R includes ten items, three of which assess optimism, three assess pessimism and the remaining four items are filling items. Optimism and pessimism do not form a continuum and should be interpreted separately [17]. Each scale ranges from 0 to 12, whereas 0 is the least pessimistic/optimistic and 12 is the most pessimistic/optimistic.

### Statistical analysis

Continuous data are presented as means ± standard deviation (SD) and were compared using Student’s *t* test. Categorical data are presented as absolute (*n*) and relative (%) frequencies and were compared using the Chi-squared test of independence or, for low values of n, Fisher’s exact test. In accordance with previous reports [6], changes in SAQ subscales were grouped into: “large decrease (> 20points)”, “moderate decrease (> 10–20 points)”, “no change (− 10 to + 10 points)”, “moderate increase (> 10–20 points)”, and “large increase (> 20 points)”. Logistic regressions were used to assess possible predictors of a large increase (> 20 points) 6 months after PCI of each SAQ subscale. Odds ratios (OR) and 95%-confidence intervals (95% CI) are reported as effect estimates.

Statistical significance was defined as *p* ≤ 0.05. Statistical analysis was performed using SPSS (SPSS Statistics for Windows, Version 28.0, IBM Corp., Armonk, NY, USA) and GraphPad Prism (Version 6.01 for Windows, GraphPad Software, La Jolla, CA, USA).

## Results

### Study population

A total of 145 patients were included in the study. Patient characteristics are shown in Table 1. The mean age was 69.9 ± 9.5 years, 47 (32.4%) patients were female, and the mean body-mass index was 28.6 ± 4.2 kg/m^2^. Patients had a mildly reduced ejection fraction (48.6 ± 7.1%). Half of the patients had three-vessel disease (51.0%), and a quarter (24.5%) of patients had either one- or two-vessel disease. Patients presented with typical comorbidities such as arterial hypertension (87.6%) and diabetes mellitus (31.0%).

**Table 1:** Patient characteristics

		*n* = 145
Age (years), mean ± SD	69.9 ± 9.5	
Male, *n* (%)	98 (68.6%)	
Body mass index (kg/m^2^), mean ± SD	28.6 ± 4.2	
Never smoking, *n* (%)		70 (48.3%)
Currently smoking, *n* (%)		18 (12.4%)
Ejection fraction (%), mean ± SD	58.6 ± 7.1	
Systolic blood pressure (mmHg), mean ± SD	136.7 ± 19.1	
Heart rate (1/min), mean ± SD	70.7 ± 11.2	
Duration of angina pectoris (months), median (IQR)	3.0 (1.0; 6.0)	
SAQ quality of life, mean ± SD	39.3 ± 19.2	
SAQ physical limitation, mean ± SD	52.7 ± 21.7	
SAQ angina frequency, mean ± SD	58.3 ± 17.6	
Number of involved vessels		
One-vessel disease, *n* (%)		35 (24.5%)
Two-vessel disease, *n* (%)		35 (24.5%)
Three-vessel disease, *n* (%)		73 (51.0%)
Total stent length (mm), mean ± SD	35.7 ± 24.8	
Maximum stent diameter (mm), mean ± SD	3.1 ± 0.5	
Typical angina pectoris, *n* (%)		125 (86.2%)
Previous PCI, *n* (%)		71 (49%)
Previous myocardial infarction, *n* (%)		39 (26.9%)
Arterial hypertension, *n* (%)		127 (87.6%)
Diabetes mellitus, *n* (%)		45 (31.0%)
Beta blocker at baseline		89 (61.4%)
Calcium channel blocker at baseline		36 (24.8%)
Nitrate at baseline		22 (15.2%)
Ranolazine at baseline		9 (6.2%)
Ivabradine at baseline		2 (1.4%)
Beta blocker at discharge		92 (63.4%)
Calcium channel blocker at discharge		43 (29.7%)
Nitrate at discharge		9 (6.2%)
Ranolazine at discharge		7 (4.8%)
Ivabradine at discharge		3 (2.1%)

*PCI* percutaneous coronary intervention, *SAQ* Seattle Angina Questionnaire

Most patients (107, 74%) received at least one antianginal drug (i.e., β-blocker, calcium channel blocker, nitrate, ranolazine, ivabradine) at baseline (Table 1). At discharge, the number of patients receiving β-blockers (89 vs 92) or calcium channel blockers (36 vs 43) increased. The number of patients receiving nitrates (22 vs 9) or ranolazine (9 vs 7) decreased.

### PCI substantially reduces symptom load and improves of quality of life

**Fig. 1:**
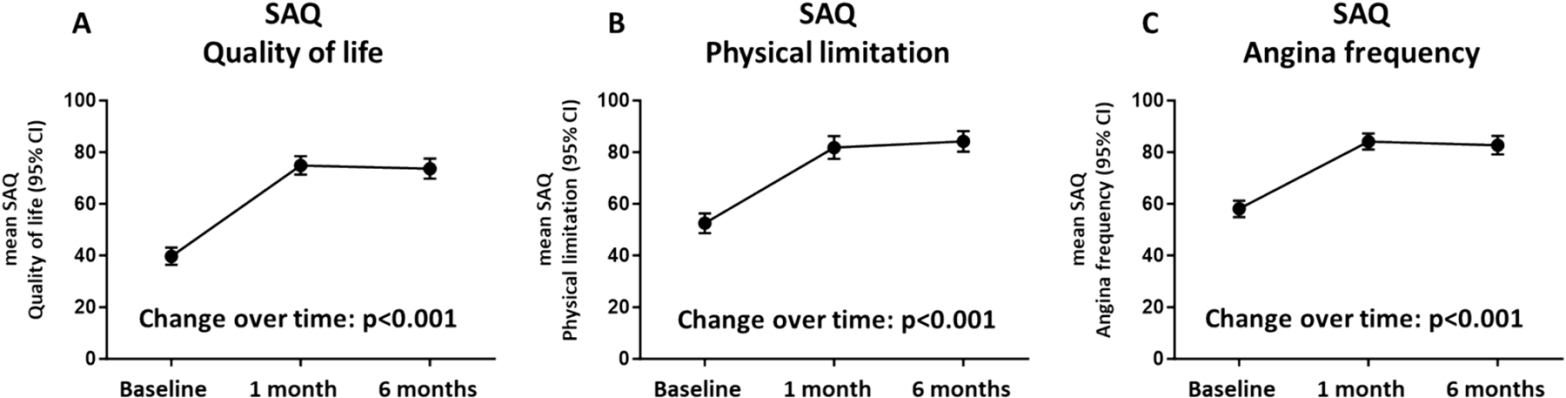
Changes in the SAQ subscales 1 and 6 months after PCI for **A** quality of life, **B** physical limitation, and **C** angina frequency. *PCI* percutaneous coronary intervention, *SAQ* Seattle Angina Questionnaire

**Fig. 2:**
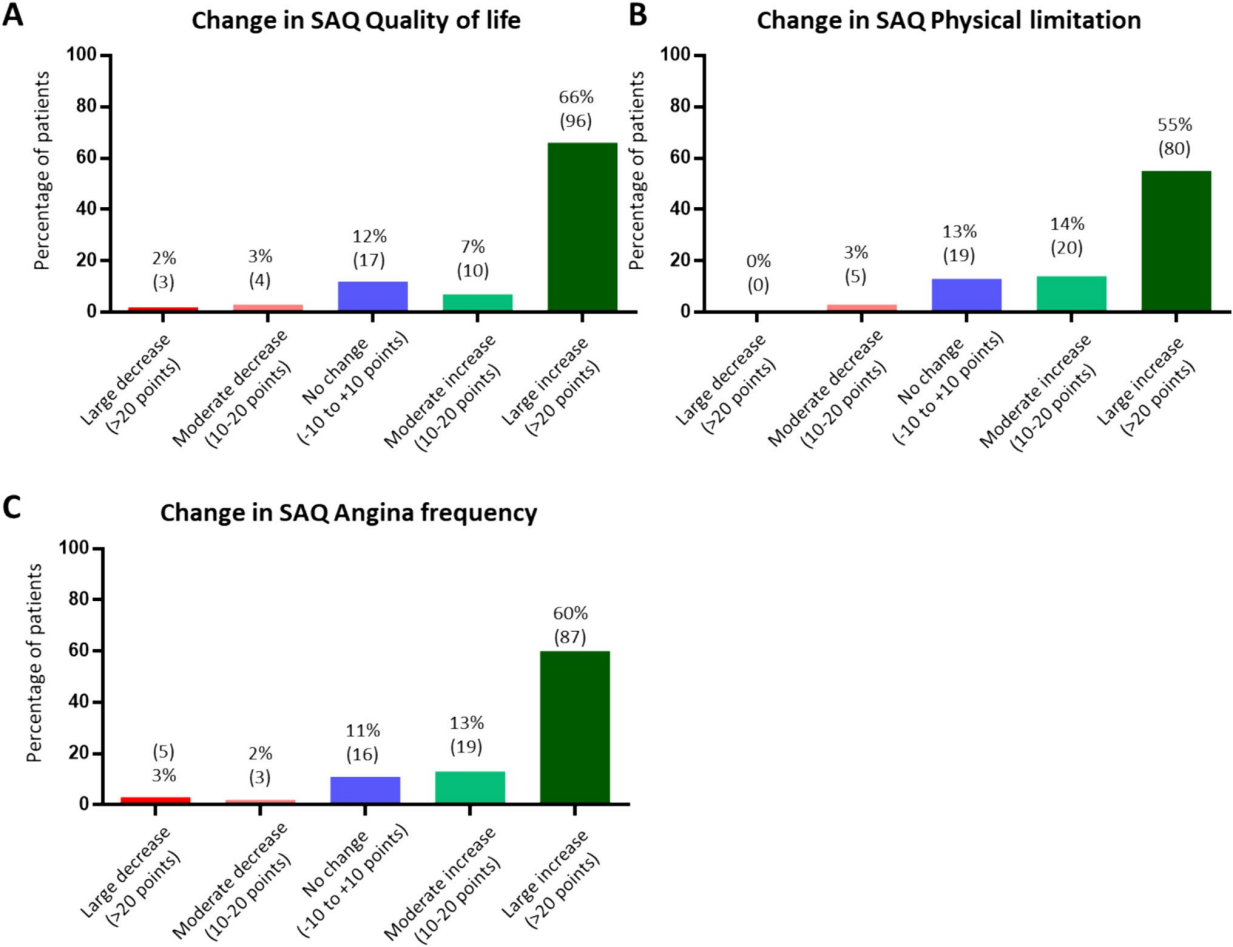
Proportion of patients experiencing large, moderate, or no changes in the SAQ subscales for **A** quality of life, **B** physical limitation, and **C** angina frequency six months after PCI. *PCI* percutaneous coronary intervention, *SAQ* Seattle Angina Questionnaire

Patients had a substantial and highly clinically relevant short- and mid-term improvement in symptom load and quality of life. The SAQ subscale score for quality of life improved from 39.7 ± 3.3 to 74.9 ± 3.5 points and 73.6 ± 3.8 points 1 and 6 months after PCI, respectively (Fig. 1A). The SAQ subscale score for physical limitation improved from 52.5 ± 3.8 to 81.8 ± 4.3 points and 84.2 ± 4.0 points 1 and 6 months after PCI, respectively (Fig. 1B). The SAQ subscale score for angina frequency improved from 58.1 ± 3.2 to 84.1 ± 3.1 points and 82.7 ± 3.6 points 1 and 6 months after PCI, respectively (Fig. 1C). Changes in each SAQ subscale score can be roughly translated to different categories of clinical influence on everyday life [6]. A difference of > 20 points denotes a large change, a difference of > 10–20 points a moderate change, and a difference of -10 to + 10 points does not translate to relevant change. Figure 2 shows that the vast majority of patients experienced a large improvement in quality of life (Fig. 2A), physical limitation (Fig. 2B), and angina frequency (Fig. 2C) 6 months after PCI. Only a few (≤ 5%) patients had worse scores 6 months after PCI.

### Predictors of improvement in symptom load and quality of life after PCI

Logistic regression analyses were performed to evaluate important demographic, clinical, procedure-related, and psychologic cofactors as predictors of a large increase in SAQ subscale scores (> 20 points, [6]) for quality of life (Table 2), physical limitation (Table 3), and angina frequency (Table 4).

**Table 2:** Predictors of a large improvement in quality of life 6 months after PCI^a^

	SAQ quality of life	
	OR (95% CI)	*p* value^b^
Age (years)	0.96 (0.92; 1.01)	0.088
Male sex	1.05 (0.46; 2.43)	0.905
Body-mass index (kg/m^2^)	1.03 (0.94; 1.14)	0.531
Canadian Cardiovascular Society class	1.70 (0.76; 3.81)	0.197
Single-vessel disease	1.06 (0.42; 2.65)	0.907
Atypical angina pectoris	1.10 (0.33; 3.64)	0.881
Angina pectoris duration (months)	1.02 (0.96; 1.08)	0.536
Previous PCI	**0.41 (0.18; 0.92)**	**0.030**
LDL cholesterol (mg/dL)	1.00 (0.99; 1.01)	0.424
Hemoglobin (mg/dL)	1.09 (0.86; 1.37)	0.482
Diabetes mellitus	0.53 (0.23; 1.20)	0.129
Renal insufficiency	**0.40 (0.17; 0.96)**	**0.040**
Ejection fraction (%)	0.97 (0.91; 1.03)	0.300
Total stent length (mm)	1.02 (1.00; 1.03)	0.143
Optimism (LOT-R points)	1.00 (0.85; 1.18)	0.981
Pessimism (LOT-R points)	0.98 (0.85; 1.14)	0.836
SAQ quality of life baseline (points)	**0.95 (0.92; 0.97)**	** < 0.001**
SAQ physical limitation baseline (points)	1.01 (0.99; 1.03)	0.342
SAQ angina frequency baseline (points)	1.02 (0.10; 1.04)	0.076

^a^Improvement was defined as a > 20-point increase on the SAQ physical limitation subscale

^b^Bold values signify statistical significance *p* < 0.05

*LDL* low-density lipoprotein, *LOT-R* revised life orientation test, *PCI* percutaneous coronary intervention, *SAQ* Seattle Angina Questionnaire

**Table 3:** Predictors of a large increase in physical limitation 6 months after PCI^a^

	SAQ Physical limitation	
	OR (95% CI)	*p* value^b^
Age (years)	1.00 (0.96; 1.04)	0.814
Male sex	0.92 (0.41; 2.06)	0.844
Body-mass index (kg/m^2^)	1.02 (0.93; 1.11)	0.749
CCS class	1.79 (0.85; 3.77)	0.126
Single vessel disease	0.60 (0.26; 1.40)	0.239
Atypical angina pectoris	**0.18 (0.06; 0.55)**	**0.003**
Angina pectoris duration (months)	0.96 (0.91; 1.01)	0.086
Previous PCI	1.10 (0.53; 2.29)	0.809
LDL cholesterol (mg/dL)	1.00 (0.99; 1.01)	0.687
Hemoglobin (mg/dL)	1.03 (0.83; 1.28)	0.796
Diabetes mellitus	1.08 (0.49; 2.42)	0.844
Renal insufficiency	0.47 (0.20; 1.13)	0.092
Ejection fraction (%)	1.00 (0.94; 1.06)	0.935
Total stent length (mm)	1.02 (1.00; 1.04)	0.070
Optimism (LOT-R points)	1.05 (0.90; 1.24)	0.515
Pessimism (LOT-R points)	**1.23 (1.06; 1.44)**	**0.008**
SAQ quality of life baseline (points)	**0.98 (0.96; 1.00)**	**0.031**
SAQ physical limitation baseline (points)	**0.95 (0.93; 0.97)**	** < 0.001**
SAQ angina frequency baseline (points)	1.00 (0.98; 1.02)	0.734

^a^Improvement was defined as a > 20-point increase on the SAQ physical limitation subscale

^b^Bold values signify statistical significance *p* < 0.05

*LDL* low-density lipoprotein, *LOT-R* revised life orientation test, *PCI* percutaneous coronary intervention, *SAQ* Seattle Angina Questionnaire

**Table 4:** Predictors of a large improvement in angina frequency 6 months after PCI^a^

	SAQ Angina frequency	
	OR (95% CI)	*p* value^b^
Age (years)	0.99 (0.95; 1.03)	0.698
Male sex	1.47 (0.68; 3.19)	0.329
Body-mass index (kg/m^2^)	0.95 (0.87; 1.05)	0.313
CCS class	0.73 (0.34; 1.54)	0.405
Single vessel disease	1.28 (0.53; 3.08)	0.585
Atypical angina pectoris	0.50 (0.18; 1.40)	0.185
Angina pectoris duration (months)	0.96 (0.91; 1.01)	0.112
Previous PCI	0.85 (0.41; 1.77)	0.665
LDL cholesterol (mg/dL)	1.01 (1.00; 1.01)	0.351
Hemoglobin (mg/dL)	0.97 (0.78; 1.21)	0.772
Diabetes mellitus	0.47 (0.22; 1.02)	0.056
Renal insufficiency	0.44 (0.19; 1.02)	0.056
Ejection fraction (%)	0.96 (0.90; 1.02)	0.156
Total stent length (mm)	0.99 (0.98; 1.01)	0.423
Optimism (LOT-R points)	1.19 (0.99; 1.42)	0.058
Pessimism (LOT-R points)	1.01 (0.88; 1.17)	0.849
SAQ quality of life baseline (points)	1.00 (0.98; 1.01)	0.633
SAQ physical limitation baseline (points)	1.01 (0.99; 1.03)	0.191
SAQ angina frequency baseline (points)	**0.96 (0.94; 0.99)**	**0.006**

^a^Improvement was defined as a > 20-point increase on the SAQ physical limitation subscale

^b^Bold values signify statistical significance *p* < 0.05

*LDL* low-density lipoprotein, *LOT-R* revised life orientation test, *PCI* percutaneous coronary intervention, *SAQ* Seattle Angina Questionnaire

For quality of life, we found that previous PCI (OR 0.41 [0.18; 0.92]) and renal insufficiency (OR 0.40 [0.17; 0.96]) were predictors of less improvement after PCI. For physical limitation, we found that the presence of atypical AP is a negative predictor of symptom improvement after PCI (OR 0.02 [0.06; 0.55]). Higher rates of pessimism were predictive of a large improvement in physical limitation (OR 1.23 [1.06; 1.44]).

Important demographic factors (age, sex, body-mass index), cardiac function (ejection fraction), comorbidities (diabetes mellitus), laboratory parameters (hemoglobin, LDL cholesterol), and coronary artery disease characteristics (Canadian Cardiovascular Society class, single- vs multi-vessel disease) were not predictive of large improvements in quality of life, physical limitation, or angina frequency. The number of antianginal drugs at discharge did not influence changes in quality of life, physical limitation, or angina frequency (Supplementary Table S1). However, for all three subscales, a lower baseline values of each subscale constitute a strong predictor of improvement.

Elevated blood pressure reduces diastolic blood flow and therefore can be a cause for or aggravate AP. At the time of measurement, 61 patients (42%) had an elevated systolic blood pressure of > 140 mmHg. However, it has to acknowledged that these measurements were likely influenced by nervosity before the catheterization and white coat hypertension and are thus probably not representative of the daily blood pressure of these patients. We therefore did not perform further analyses stratified by our spot measurements of blood pressure. However, the observation that more patients received β-blockers (92 vs 89) and calcium channel blockers (43 vs 36) at discharge than at baseline (Table 1) may be due to poorly controlled blood pressure in these individuals.

### Patient beliefs about symptom improvement after PCI

In addition to the SAQ, patients were asked simple qualitative questions about their behavior and expectations both at baseline and at the follow-up assessments.

Before PCI, the vast majority of patients were optimistic about the positive effects of PCI as only 3 (2%) answered the question “Do you think that PCI will help you?” negatively, whereas 140 (98%) answered it positively. At the 6 month follow-up, most patients still answered the question “Did the PCI help you?” positively (*n* = 118, 89%) (Supplementary Table S2). There was a strong correlation between the improvement in quality of life, physical limitation, and angina frequency, and a positive answer to this question “Did the PCI help you?” 6 months after PCI. Patients who answered this positively had less physical limitation and a lower angina frequency at baseline. Most patients answered the question “Did the medication help you?” positively (*n* = 101, 77%) (Supplementary Table S3). These patients experienced a larger improvement for quality of life.

Patients who answered the question “Do you exercise more often than before the PCI?” positively (*n* = 81, 60%) experienced a much larger and highly clinically relevant improvement in quality of life after PCI than patients who answered this question negatively (*n* = 53, 40%) (Table 5). There were no differences in baseline quality of life or for the other SAQ subscales physical limitations or angina frequency between these two groups. There were no differences between these two groups regarding demographic parameters (age, body-mass index, sex), ejection fraction, or PCI parameters (total stent length, maximal stent diameter).

**Table 5:** Patient characteristics and quality of life after 6 months according to their answer to the question: “Do you exercise more often than before PCI?”

	Do you exercise more than before PCI?		
	Yes (*n* = 53)	No (*n* = 81)	*p* value^a^
Age (years)	69.3 ± 9.0	70.9 ± 9.5	0.356^T^
Body-mass index (kg/m^2^)	28.6 ± 4.0	28.7 ± 4.1	0.871^T^
Male sex (%)	67.9%	69.1%	0.883^Chi^
Ejection fraction (%)	57.9 ± 7.3	59.1 ± 7.1	0.369^T^
Total stent length (mm)	37.6 ± 26.0	35.9 ± 24.7	0.707^T^
Maximal stent diameter (mm)	3.2 ± 0.5	3.1 ± 0.5	0.426^T^
Quality of life before PCI (SAQ subscale points)	37.3 ± 17.6	41.3 ± 20.3	0.243^T^
Physical limitation before PCI (SAQ subscale points)	56.4 ± 19.6	50.2 ± 22.5	0.108^T^
Angina frequency before PCI (SAQ subscale points)	58.1 ± 15.3	58.4 ± 19.3	0.929^T^
Difference in quality of life 6 months after PCI (SAQ subscale points)	42.5 ± 22.0	28.7 ± 26.1	**0.002**^**T**^
Difference in physical limitation 6 months after PCI (SAQ subscale points)	33.0 ± 20.8	30.8 ± 25.0	0.606^T^
Difference in angina frequency 6 months after PCI (SAQ subscale points)	28.3 ± 21.9	22.5 ± 23.6	0.156^T^
Optimism (LOT-R subscale points)	8.3 ± 3.3	8.0 ± 2.6	0.687^T^
Pessimism (LOT-R subscale points)	5.9 ± 3.3	5.3 ± 2.3	0.314^T^

^a^Bold *p* values highlight statistical significance *p* < 0.05

^Chi^Chi-squared test

^T^Student’s *t* test

*LOT-R* revised life orientation test, *PCI* percutaneous coronary intervention, *SAQ* Seattle Angina Questionnaire

## Discussion

Our analysis shows that (1) in patients with symptomatic CCS PCI leads to a quick and sustained reduction in symptom load and improvement in quality of life, (2) high initial-symptom load is a strong predictor of symptom improvement after PCI, whereas demographic and clinical factors did not relevantly influence symptomatic outcome, and (3) increased daily exercise strongly correlates with a larger increase in quality of life 6 months after PCI.

### PCI provides quick and sustained improvement in symptom load and quality of life

Many studies have indicated that PCI provides a highly significant improvement in symptom load and quality of life in patients with CCS which is also reflected in the current European Society of Cardiology [1] and American Heart Association [18] guidelines. The recent ORBITA-2 trial is the first controlled and randomized trial to prove that PCI reduces angina symptoms in patients with symptomatic CCS [5]. Rajkumar et al. included only patients with AP and found that 3 months after PCI, 40% of patients in the PCI group and only 15% in the placebo group were angina-free [5]. Initial-symptom load and the magnitude of the improvement were similar to our findings. The ISCHEMIA trial included patients with CCS and relevant ischemia [19]. A third of these patients did not have angina symptoms at baseline which is reflected in the much higher baseline SAQ scores compared to those in our study. The trial compared unblinded invasive with conservative treatment. PCI provided a quick and sustained improvement in physical limitation, angina frequency, and quality of life for up to 4 years [19]. Our study adds to these findings as it shows that patients with symptomatic CCS experience a considerable improvement in symptom load and quality of life 1 and 6 months after PCI.

### High initial-symptom load and low quality of life are the strongest predictors of better symptom outcome after PCI

Although there is no clear survival benefit from either conservative or invasive therapy in CCS, symptom control is an important goal. The ORBITA trial showed that on the background of optimal medical treatment, additional PCI does not provide a benefit in terms of symptoms [3]. However, as discussed above, PCI can be a potent therapy to reduce symptom load. Therefore, optimal patient selection for invasive treatment is crucial, and the assessment of predictors of optimal symptom outcomes is warranted. Arnold et al. found that in patients with stable AP, unstable AP, or NSTEMI who underwent PCI, the rates of prior myocardial infarction, prior PCI, prior coronary artery bypass grafting, and chronic heart failure were higher in the group with persisting AP [7], showing that patients with preexisting coronary artery disease are less likely to be angina-free after PCI. The study by Arnold provides a prediction model for symptom improvement after PCI. Spertus et al. investigated predictors of quality-of-life benefit in patients after PCI [6]. They found that patient characteristics (demographic parameters, comorbidities) and disease severity (number of diseased vessels, ejection fraction) accounted for only a very small proportion (roughly 2%) of the predictive value for an increase in quality of life 12 months after PCI. In contrast, lower baseline health status as assessed by the SAQ parameters physical limitation and angina frequency provided a good estimate of improved quality of life (approximately 22%) [6]. The study excluded patients with acute myocardial infarction but did not distinguish between instable and stable CCS. It should be taken into consideration that both medical and invasive therapy have evolved considerably since the study was conducted in 2004 which limits the generalizability of these results on present-day patients with CCS. The analysis of the ISCHEMIA trial data for symptom improvements in patients with CCS and ischemia by Spertus et al. shows that worse baseline health status (i.e., more frequent and more severe AP) predicts larger symptom improvements; however, a third of patients in ISCHEMIA were asymptomatic [19]. Our analysis concentrates on patients with symptomatic CCS. In accordance with previous findings we identified worse baseline health status as the most important predictor of improvements in symptoms and quality of life. However, this seems to be almost self-explanatory as patients with only a small symptom load or high initial quality of life simply cannot improve much further in each realm. It is surprising that other important cofactors such as demographic parameters or comorbidities did not seem to influence the success of PCI in treating the symptoms of these patients. Taken together with findings from previous studies, our data lead to two main conclusions: first, that PCI should be preferentially targeted at patients with high symptom load and low quality of life; second, in the absence of evidence for effects on PCI, certain demographic and clinical parameters such as age, body-mass index and comorbidities should not be used to select patients for invasive treatment.

### Influence of patient beliefs and exercise on symptom outcome after PCI

Patient beliefs and expectations are powerful modulators of outcome especially of symptom relief of therapeutic interventions [20]. A prominent example is the placebo effect that can be partly explained by these factors [20]. It is difficult to establish a clear cause-effect relationship for the intricate interplay between psychologic traits, patient beliefs and expectations, and symptom load. However, important clues can be derived from correlation. Arnold et al. found that in patients with PCI (stable AP, unstable AP, NSTEMI), the rate of depression was higher in the group with persisting AP than in the group that was angina-free 6 months after the procedure [7]. We investigated the effect of the personality traits optimism and pessimism on the changes in symptoms after PCI. To our knowledge, this has not been included in trials investigating the effects of PCI in patients with CCS. We hypothesized that patients with higher optimism and lower pessimism would be more susceptible to the “placebo effect" and therefore experience a larger benefit in terms of symptoms. However, we did not find such a connection. On the contrary, patients with more pessimism were more likely to have a large increase in their physical limitation score. Nevertheless, the amount of optimism or pessimism had no effect on the change in quality of life or angina frequency. Therefore, the effect on physical limitation has to be interpreted with caution. We do not think that our data support our hypothesis that optimism or pessimism are important modulators of effect of PCI on symptoms and quality of life.

We also asked the patients simple binary questions about their expectations before PCI and their perceived benefits after PCI. Almost all patients had the positive expectation that PCI will improve their symptoms. This is not surprising because without these expectations patients would probably not consent to this procedure. 6 months after PCI, most patients thought that PCI helped them. This correlated well with more detailed improvements as measured with the SAQ. Therefore, asking the simple question “Did PCI help you?” provides the physician with valid information about the health status and quality of life of the patient after PCI. We also asked the patients if they exercised more after PCI. Interestingly, the patients who said that they did exercise more than before PCI had a significantly larger increase in quality of life than patients that did not, although there were no differences in improvements in physical limitation or angina frequency between the two groups. In addition, demographic parameters, cardiac health, and comorbidities were the same in the two groups. No causality can be derived from this observation, however, as physical limitations and angina frequency did not differ between groups, it seems that the larger increase in quality of life could be attributable to more physical exercise instead of better physical health status. Physical activity and sport are important factors for primary and secondary prevention especially in cardiovascular health [1, 18, 21]. In addition, they are both linked to psychologic well-being [22]. In our analysis, physical activity led to a 50% higher increase in quality of life independent from actual physical status. This is highly relevant as quality of life is a paramount goal in patient-centred medicine.

## Limitations

This current analysis is a secondary analysis of a randomized controlled trial that was not specifically powered for the present analysis. Therefore, generalizability is limited. As there was no sham control for PCI, the improvements after PCI may not all be attributable to the procedure. The general limitations in using patient-reported outcome measures such as the SAQ and the LOT-R are also applicable in our analysis (e.g., subjectivity, bias, variability, external validity).

## Conclusions

Our analysis shows that PCI leads to a quick and sustained reduction in symptom load and improvement in quality of life in patients with symptomatic CCS. The best predictor for symptom improvement after PCI is high initial-symptom load. In contrast, demographic and clinical factors did not relevantly influence symptomatic outcome. Interestingly, increased daily exercise strongly correlates with a larger increase in quality of life 6 months after PCI.

## Data Availability

Anonymized study data is available upon reasonable request.

## Abbreviations

AP: Angina pectoris
CCS: Chronic coronary syndrome
CCS class: Canadian Cardiovascular Society grading score
LOT-R: Revised life orientation test
NSTEMI: Non-ST elevation myocardial infarction
OR: Odd’s ratio
PCI: Percutaneous coronary intervention
SAQ: Seattle Angina Questionnaire
STEMI: ST elevation myocardial infarction
